# Supplementary material for: Reliability of Self-Reported Height and Weight in Children: A School-Based Cross-Sectional Study and a Review
Source: Nutrients. 2022 Dec 23;15(1):75. doi: 10.3390/nu15010075 (PMC9824624; doi:10.3390/nu15010075)
Supplement: Supplementary file 1 [file nutrients-15-00075-s001.zip › nutrients-2079809-supplementary.pdf]

nutrients

MDPI

Reliability of Self–Reported Height and Weight in Children: A School–Based Cross–Sectional Study and a Review

Supplementary Table S1. Questionnaire.

**Child questionnaire**
**Outside of school, how many days per week do you do sports or a physical activity to the point of sweating or being out of breath ?**<br>a) Never<br>b) A few times per month<br>c) 1–2 days per week<br>d) 3–4 days per week<br>e) 5–6 days per week<br>f) Every day
**During the week, how much time do you spend in front of a TV screen on average ?**<br>a) Never<br>b) Less than 15 minutes per day<br>c) 15–30 minutes per day<br>d) 30–60 minutes per day<br>e) 1–2 hours per day<br>f) More than 2 hours per day
**Parent questionnaire**
**What is the highest level of education that you have attained**<br>a) Obligatory school<br>b) Apprenticeship<br>c) High school baccalaureate<br>d) University, college<br>e) Other
**Nationality**<br>a) Swiss<br>b) Other
**Anthropometric measures**
Before taking the measures, ask the child his weight : ………. (kg) and height ….…… (cm(.
**Measures : Child in light clothes or underwear, without shoes :**<br>**Weight (kg) :**<br>**Height (cm) :**

Supplementary Table S2. Overview of the studies identified and their key results.

	Pearson's *r* Correlation			Mean Difference			Prevalence Difference (%)		
**Study ID**	**Weight**	**Height**	**BMI**	**Weight (kg)**	**Height (cm)**	**BMI (kg/m²)**	**Overweight**	**Obese**	**Overweight and obese**
Aasvee 2015	0.95	0.95	0.89	−1.4	−0.5	−4.0			−3.6
Abalkhail 2002	0.90	0.72		−2.7	4.0				
Abraham 2004				−1.2	−0.9	−0.1			
Ambrosi−Randic 2007	0.98	0.97	0.95	−0.7	0.2				
Andersen 2005			0.91	−0.7	0.0	−0.4			
Bae 2010				−1.5	0.2	−0.6		−4.7	
Baile 2014	0.95	0.95	0.93	−0.8	0.1	−0.3	−2.2	−1.1	−3.3
Beck 2012	0.89	0.65	0.79	−3.4	−0.1	−1.9			
Beghin 2013				−0.8	0.7				
Berg 2001				−0.4	0.3	−0.2			
Brault 2015	0.98	0.92	0.90	−1.0	−0.8	−0.2	−1.5	−0.1	−1.6
Brener 2003	0.93	0.90	0.89	−3.5	2.7	−2.6			−11.1
Brettschneider 2015				−0.7	0.3	−0.3	−1.3	−1.3	−2.6
Brooks−Gunn 1987	0.98	0.75		−1.2	1.4				
Buttenheim 2013						−1.2	1.0	−9.0	−8.0
Chan 2013				−0.6	−0.2	−0.2			
Charalampos 2009	0.94	0.86	0.89	−0.7	1.3	−0.6	−3.3	−1.0	
Chau 2013	0.98	0.96	0.93	−0.9	−0.4	−0.3	−2.1	−1.3	
Clarke 2014				−0.6					
Dalton 2014									4.6
Davis 1994	0.94	0.86	0.86						
De Vriendt 2009				−0.7	−0.1	−0.2	−2.0	−0.8	
Domingues 2011			0.84				−3.7	−1.9	
Drake 2013								−6.2	−4.9
Ekström 2015				−1.1	0.5	−0.5	0.0	0.0	
Elgar 2005	0.95	0.82	0.88	−0.5	0.4	−0.3	−4.8	−1.6	
Enes 2009				−1.0	−2.5	0.1			
Farre Rovira 2002						−0.9			
Fonseca 2010	0.96	0.89	0.85	−1.0	0.7	−0.6	−0.5	−1.1	
Fortenberry 1992				−1.4	0.5				−0.3
Frayon 2017				−3.3	−2.9	−0.4			

	Pearson's *r* Correlation			Mean Difference			Prevalence Difference (%)		
**Study ID**	**Weight**	**Height**	**BMI**	**Weight (kg)**	**Height (cm)**	**BMI (kg/m²)**	**Overweight**	**Obese**	**Overweight and obese**
Ghosh−Dastidar 2016				−1.9	−0.9	−0.5	−2.1	−2.1	−4.2
Giacchi 1998	0.94	0.95		−1.8	1.4	−1.0	−7.5		
Goodman 2000	0.95	0.94	0.92	−0.6		−0.1		−1.6	
Hauck 1995	0.93	0.73	0.84	−1.8	1.0				−6.0
Himes 1992	0.94	0.83	0.86	−2.7	0.5	−1.1			
Himes 2001				−0.9	−1.1	0.0			
Himes 2005	0.95	0.85	0.87	−2.7	1.8	−2.3			
Jansen 2006	0.85	0.80	0.75	−6.1	−1.5	−1.5			
Jayawardene 2014				−1.0	1.1	−0.7	−0.2	−4.1	−4.3
Kee 2017				−1.6	−1.2	−0.4	0.3	−1.0	
Kurth 2010				−0.7	0.4	−0.4			
Lee 2006	0.83	0.52	0.61	−4.1	−4.1	0.7			
Lee 2013	0.97	0.96	0.93	−0.4	0.8				−4.5
Legleye 2014			0.87	−1.6	1.3	−0.9	−6.7	−1.7	
Linhart 2010				−0.1		−0.1	0.1	−2.3	
Morrissey 2006	0.96	0.91	0.92	−1.5	−0.2	−0.4			−2.7
Ohlmer 2012				−0.5	−1.0				
Perez 2015	0.94	0.82	0.88	−0.7	−0.6	−0.4			
Rasmussen 2007	0.95	0.95	0.93	−0.8	0.1	−0.3	−2.2	−1.1	−3.3
Rasmussen 2013				−1.3	0.2				−5.7
Robinson 2014	0.88	0.32		−3.1	1.9				−1.1
Rodrigues 2013				−0.4	0.0	−0.1			
Seghers 2010							−2.2	−1.4	
Stefan 2019	0.97	0.96	0.95	−0.8	0.8	−0.5			
Strauss 1999	0.93	0.89	0.87	−0.4	−1.0				
Tienboon 1992	0.87	0.77	0.67	−0.8	0.4	−0.4			
Tokmakidis 2007	0.96	0.91	0.90	−2.1	1.4	−1.2	−5.7	−5.2	
Tsigilis 2006	0.95	0.97	0.92	−2.1	0.5	−0.9			
Wang 2002				−2.0	1.1		−13.2	−2.1	−7.0
Yoshitake 2012	0.98	0.95	0.94	−0.2	−0.4	0.0			
Zhou 2010	0.91	0.94	0.81	−2.3	−1.4	1.2			−5.1
Current study	0.96	0.92	0.88	−1.4	−0.9	−0.4			−1.9
